# Non-Protein-Coding RNA and Acute Kidney Injury: New Developments from Pathogenesis to Potential Biomarker

**DOI:** 10.3390/genes16101194

**Published:** 2025-10-13

**Authors:** Grazia Maria Virzì, Anna Clementi, Monica Zanella, Claudio Ronco

**Affiliations:** 1 Department of Nephrology, Dialysis and Transplant, San Bortolo Hospital, 36100 Vicenza, Italy; monica.zanella@aulss8.veneto.it; 2 IRRIV-International Renal Research Institute Vicenza-Foundation, 36100 Vicenza, Italy; a.clementi81@virgilio.it (A.C.); cronco@goldnet.it (C.R.); 3 Department of Nephrology and Dialysis, Santa Marta and Santa Venera Hospital, 95024 Acireale, Italy

**Keywords:** non-protein-coding RNA, microRNA, AKI, microvescicles

## Abstract

Acute Kidney Injury (AKI) is a critical medical condition characterized by a sudden and significant decline in renal function over a short timeframe. Commonly triggered by factors such as sepsis, ischemia–reperfusion injury, or nephrotoxic agents, AKI is linked to substantial rates of morbidity and mortality. In recent years, small non-coding RNAs have gained attention as promising biomarkers for the early diagnosis and potential treatment of AKI. Among them, microRNAs (miRNAs)—short RNA sequences of 21–25 nucleotides that regulate gene expression via sequence-specific binding—stand out due to their remarkable stability in biological fluids such as plasma and urine. Notably, certain miRNAs, including miR-21, miR-30, miR-494, and miR-29, have shown the ability to detect AKI earlier than traditional biomarkers like serum creatinine, offering the potential to enhance clinical decision-making. This narrative review aims to provide a comprehensive overview of the recent findings regarding the involvement of non-coding RNA, in particular microRNAs, in both the early diagnosis and therapeutic strategies for AKI. By highlighting their potential as sensitive biomarkers and novel treatment targets, this review seeks to contribute to advancing clinical approaches that improve patient outcomes. Ultimately, a deeper understanding and utilization of microRNAs could lead to the development of new diagnostic tools and targeted therapies for AKI, helping to prevent progression to chronic kidney disease and reduce associated mortality rates. However, further clinical studies and translational applications are still needed to validate these findings and implement them in patient care.

## 1. Introduction

Acute Kidney Injury (AKI) is a serious clinical condition marked by a sudden decline in kidney function in a short timeframe, often leading to disturbances in fluid balance and electrolyte levels. Common causes of AKI include sepsis, ischemia–reperfusion injury, medication-induced toxicity, urinary tract obstruction, and rhabdomyolysis [1]. AKI represents a significant global health challenge, affecting approximately 13.3 million individuals each year and resulting in over 2.3 million deaths annually. The burden is unevenly distributed, with 85% of cases occurring in low- and middle-income countries (LMICs), where resource limitations often hinder early recognition and access to life-saving interventions such as dialysis [2]. Although the prevalence of AKI among hospitalized patients has been estimated at 23.2% [3], this figure rises to 57.3% for individuals admitted to intensive care units [4]. Mortality associated with AKI varies considerably, ranging from 3.4% in stage 1 cases to 24.1% in patients who require dialysis [4]. Specifically, even one episode of AKI carries a substantial risk of death. A stage 1 AKI event occurring during critical illness has been shown to be independently linked to higher mortality over a 10-year period [5].

Furthermore, this clinical condition frequently leads to unfavorable long-term outcomes, as it can progress to chronic kidney disease (CKD) and, in some instances, irreversible end-stage renal failure (ESRD). Indeed, surviving renal tubular cells are sometimes unable to fully regenerate the injured tissue, which can lead to renal fibrosis and persistent impairment of kidney function [6].

Consequently, promptly identifying AKI is essential not only for improving the management of this condition but also for reducing the risk of subsequent complications. Early recognition enables timely interventions that may limit kidney damage and improve patient outcomes. However, despite advances in research, there is still no universally accepted gold standard for the early diagnosis of AKI, and current diagnostic methods often lack sensitivity and specificity in detecting kidney injury at its initial stages [7,8]. Over the past decade, a substantial body of research has focused on investigating the role of emerging biomarkers in the early detection and prognosis of AKI. Numerous studies have explored a wide range of promising biomarkers that could improve diagnostic accuracy, allow earlier identification of kidney injury, and help guide therapeutic decisions. Despite this growing interest and significant progress, further validation in clinical settings is still needed before these novel biomarkers can be routinely implemented in practice [9,10]. These biomarkers encompass neutrophil gelatinase-associated lipocalin (NGAL), the cysteine protease inhibitor Cystatin C, kidney injury molecule-1 (KIM-1), as well as the products of insulin-like growth factor binding protein 7 (IGFBP-7) and tissue inhibitor of metalloproteinases 2 (TIMP-2) (Nephrocheck). With reported areas under the curve (AUC) of 0.67 for NGAL [11], 0.71 for cystatin C [12], 0.65 for KIM-1 [11], and 0.80 for the products of IGFBP7 and TIMP-2 [13], these biomarkers have demonstrated superior diagnostic accuracy for AKI compared to conventional markers such as creatinine and urea. However, up until now, the majority of these biomarkers have not yet been fully integrated into routine clinical practice. Meanwhile, a growing body of research involving both animal models and human subjects has recently explored the potential role of non-coding RNAs, particularly microRNAs (miRNAs), in the diagnosis and treatment of AKI.

## 2. Advancements in Omics Technologies: A Holistic Approach to Understanding Biological Mechanisms

**Figure 1:**
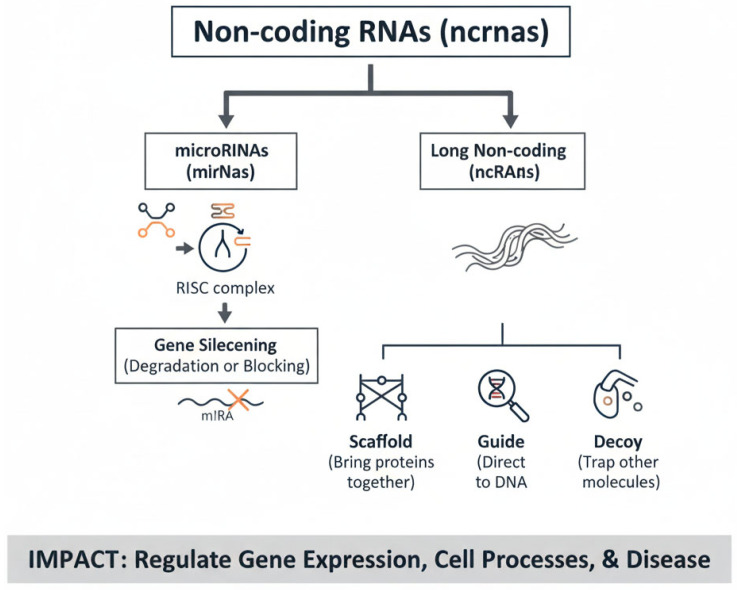
The Functional Diversity of Non-coding RNAs. This diagram provides a conceptual overview of the diverse roles played by non-coding RNAs (ncRNAs). The interconnected pathways show how the dysregulation of both miRNAs and lncRNAs can impact fundamental biological processes, ultimately contributing to the development of various diseases.

In recent years, omics technologies—such as genomics, transcriptomics, proteomics, and metabolomics—have revolutionized molecular biology by enabling comprehensive and high-throughput analyses of cellular and molecular processes at an unprecedented scale. These powerful approaches provide a holistic view of biological systems, allowing researchers to simultaneously examine thousands of genes, proteins, and metabolites in a single experiment. This level of detail has greatly enhanced our understanding of cellular functions, disease mechanisms, and biological responses to various stimuli [14,15]. For example, genomics has enabled the discovery of genetic variants linked to complex diseases, while transcriptomics has been pivotal in unraveling cell-specific gene expression signatures in cancer and immune disorders [16,17]. Proteomics has revealed dynamic protein–protein interactions and post-translational modifications critical for signaling pathways [18], and metabolomics has offered unique insights into cellular energy states, metabolic reprogramming in tumors, and biomarkers of organ injury [19]. Together, these layers of information provide complementary perspectives, making it possible to construct integrated models of health and disease. By integrating data across multiple omics layers (multi-omics), scientists can uncover complex interactions and regulatory networks that were previously hidden. Multi-omics approaches have already proven transformative in precision medicine: for instance, in oncology, combined genomic and proteomic profiling has enabled the identification of actionable mutations and the development of targeted therapies; in nephrology, metabolomic signatures are being investigated as early indicators of AKI, and in infectious diseases, transcriptome-wide analyses of host responses have shed light on pathways underlying susceptibility to pathogens. Among the groundbreaking discoveries facilitated by these technologies is the identification of non-coding RNAs (ncRNAs), a diverse group of RNA molecules that do not code for proteins but play essential roles in regulating gene expression, epigenetic modifications, and cellular communication. High-throughput sequencing technologies revealed the abundance and functional diversity of ncRNAs, including microRNAs, long non-coding RNAs (lncRNAs), and circular RNAs [20,21] (Figure 1). These molecules have been implicated in a wide spectrum of biological processes, ranging from embryonic development and tissue differentiation to the pathogenesis of cancer, cardiovascular disorders, and kidney diseases. For instance, specific microRNA profiles are now recognized as biomarkers of prognosis and therapeutic response in oncology, while lncRNAs are emerging as key regulators of fibrosis and inflammation [22]. Collectively, the integration of omics technologies has shifted biomedical research from a reductionist to a systems-level perspective, paving the way toward personalized medicine, where diagnosis, prognosis, and therapy can be tailored based on individual molecular profiles.

## 3. Aim of This Work

This narrative review aims to provide a comprehensive overview of the recent findings regarding the involvement of non-coding RNA, in particular microRNAs, in both the early diagnosis and therapeutic strategies for AKI. By highlighting their potential as sensitive biomarkers and novel treatment targets, the review seeks to contribute to advancing clinical approaches that improve patient outcomes. Ultimately, better understanding and utilizing microRNAs could enhance the management of AKI, help prevent progression to chronic kidney disease, and reduce associated mortality rates. This review could serve as a concise and accessible guide for clinicians and researchers approaching the field of non-coding RNAs in the context of acute kidney injury (AKI), particularly from a nephrology and critical care perspective. In this way, our manuscript aims to bridge the gap between basic research and clinical nephrology, especially for those who are not yet familiar with the non-coding RNA landscape.

## 4. Materials and Methods

A comprehensive literature search using the PubMed and Cochrane databases is essential for conducting a thorough and accurate systematic review of the topic. Employing well-designed and specific search strings ensures that relevant studies are effectively identified, minimizing the risk of missing important data. Careful selection and screening of articles based on predefined inclusion and exclusion criteria further guarantee the quality and relevance of the evidence included in this narrative review, ultimately strengthening the validity and reliability of the conclusions drawn. In this process, case reports and case series were excluded due to their limited generalizability, while greater emphasis was placed on experimental studies and epidemiological research, which provide stronger formal evidence and more robust data for analysis. Additionally, we excluded publications not written in English or those for which only the abstract was available in English. Conference abstracts, editorials, commentaries, and non-peer-reviewed sources were also excluded to ensure that only rigorously evaluated and scientifically sound studies were included. This stringent selection process was instrumental in minimizing bias and enhancing the overall scientific rigor of the review. The literature search included studies published in English, encompassing in vitro studies, animal studies, observational studies, and both clinical and preclinical research. The search covered publications from 2009 to 2024 to ensure a consistent and relevant data set. The first half of 2025 was excluded from the analysis. In practice, PubMed and Cochrane databases served as the main sources for identifying relevant studies for this review. A comprehensive literature search was conducted using the PubMed and Cochrane databases with the following search terms: (acute kidney injury OR acute kidney damage) AND (microRNAs OR miRNAs), as well as (acute kidney injury OR acute kidney damage) AND (non-coding RNAs). Additionally, PubMed was extensively queried with more specific keywords to further refine and enrich the findings included in this review. References cited within the selected articles were also examined to identify additional relevant studies. This comprehensive approach helped to gather a comprehensive and focused body of literature for the review.

## 5. Non-Protein-Coding RNA and Its Family

In recent years, a growing body of research has highlighted the crucial role of non-coding RNAs (ncRNAs), particularly microRNAs (miRNAs), in the underlying mechanisms of AKI. These small RNA molecules have been shown to regulate gene expression involved in inflammation, cell death, and tissue repair processes that contribute to the onset and progression of AKI. Understanding the complex functions of ncRNAs in kidney injury not only sheds light on disease pathophysiology but also opens new avenues for developing innovative diagnostic biomarkers and targeted therapeutic strategies [21,22,23].

The completion of the human genome sequencing project in 2001 marked a pivotal milestone in biomedical research, initiating extensive efforts to understand the function of human genes. Initially, scientists believed that the majority of the human genome consisted of protein-coding genes responsible for producing the proteins essential to cellular function. However, a major breakthrough came with the discovery that only about 2% of the genome actually encodes proteins. This finding fundamentally changed our understanding of genomic architecture, highlighting the vast proportion of the genome composed of non-coding regions whose functions were previously unknown. This revelation has since driven intense research into the roles of non-coding elements, such as regulatory RNAs and other genomic sequences, reshaping our view of gene regulation and complexity in human biology. However, research has demonstrated that approximately 80% of the human genome is actively transcribed into RNA molecules, far exceeding the proportion of protein-coding sequences [23,24]. The ENCODE Project Consortium 16 provided comprehensive evidence that the vast majority of these transcripts do not translate into proteins. Instead, they belong to a diverse group of non-protein-coding RNAs, which play critical regulatory roles in gene expression, chromatin structure, and cellular function. This discovery has shifted the focus of genomic research toward understanding the various classes of ncRNAs—including microRNAs, long non-coding RNAs, and others—and their involvement in health and disease, highlighting their potential as diagnostic markers and therapeutic targets [21,25]. The family of ncRNAs represents a large and heterogeneous group of RNA molecules that perform a wide array of regulatory and structural functions within the cell [13]. By widely accepted convention, an ncRNA is classified as either long or short based on its length, with the cutoff typically set at 200 nucleotides. RNAs longer than this threshold are defined as long non-coding RNAs (lncRNAs), while those shorter are considered small non-coding RNAs (sncRNAs). The small ncRNA category includes various subtypes such as small nuclear RNA (snRNA), small nucleolar RNA (snoRNA), repeat-associated small interfering RNA (rasiRNA), small cytoplasmic osteosarcoma RNA (scoRNA), microRNA (miRNA), small interfering RNA (siRNA), piwi-interacting RNA (piRNA), and transcription initiation RNA (tiRNA) [21,25,26] (Table 1).

Among these, microRNAs are typically short molecules, usually comprising fewer than 25 nucleotides, and are extensively studied for their crucial roles in post-transcriptional gene regulation through mRNA targeting and silencing. This classification framework helps to organize the diverse ncRNA species according to their size and biological function, facilitating more targeted research into their specific roles in cellular processes and disease mechanisms.

## 6. Fundamentals of microRNA Function and Their Role in Cellular Regulation

MicroRNAs (miRNAs) are small non-coding RNA molecules, typically 21 to 25 nucleotides in length, that play a key role in regulating gene expression. They exert their function through sequence-specific recognition and binding, primarily targeting the 3ʹ untranslated region (3′UTR) or, less commonly, the 5ʹ untranslated region (5ʹUTR) of messenger RNA (mRNA) transcripts. In some cases, miRNAs can also interact with promoter regions of genes. In binding to these specific sequences, miRNAs modulate gene expression mainly through post-transcriptional mechanisms, such as inducing mRNA degradation or inhibiting translation. This fine-tuning of mRNA levels allows miRNAs to influence diverse biological processes, including development, cell differentiation, apoptosis, and stress responses, and their dysregulation has been implicated in numerous diseases [23,27,28,29,30].

MiRNAs originate from primary nuclear transcripts, known as long primary transcripts of miRNAs (pri-miRNAs), which are synthesized in the nucleus by RNA polymerase II. These long transcripts undergo initial processing by the RNase III enzyme Drosha, which cleaves them into precursor miRNAs (pre-miRNAs) approximately 70 to 100 nucleotides in length. The pre-miRNAs are then transported from the nucleus to the cytoplasm, where they undergo further cleavage by another RNase III enzyme, Dicer, resulting in the formation of mature miRNA duplexes composed of double-stranded RNA molecules. One strand of this duplex is subsequently incorporated into the RNA-induced silencing complex (RISC) to mediate gene silencing [31].

In addition to the canonical steps of miRNA biogenesis, several regulatory checkpoints exist that modulate transcription, processing, and maturation of miRNAs in response to cellular signals. A key example is p53, which not only transactivates miRNA genes such as miR-34, miR-145, miR-192/215, and miR-107, but also enhances post-transcriptional processing of primary miRNAs via the Drosha microprocessor complex through interaction with DEAD-box helicases p68/p72 in response to DNA damage [32,33]. Conversely, mutant forms of p53 impair miRNA maturation by disrupting the association between Drosha and its cofactors, leading to reduced processing of specific tumor-suppressive miRNAs [32,33]. Another oncogenic transcription factor, c-Myc, similarly influences biogenesis at multiple levels: it directly activates transcription of miRNA clusters (e.g., miR-17-92), represses many others, and importantly upregulates the expression of Drosha itself by binding to its promoter E-box elements, thereby increasing the capacity of the processing machinery [34,35]. Together, these mechanisms show that miRNA expression is finely tuned by transcription factors both at the level of gene transcription and of processing efficiency, integrating stress, developmental, and oncogenic signals.

MiRNAs have been demonstrated to regulate the expression of numerous genes by binding to the 3′-untranslated regions (3′UTRs) of their target mRNAs, thereby either suppressing or, in some contexts, enhancing gene expression. In any given cell type, a combination of cell-specific and broadly expressed miRNAs can simultaneously influence the translation and stability of multiple mRNA transcripts. Beyond their intracellular functions, miRNAs can also be actively secreted into the circulation, where they are transported within extracellular vesicles or associated with RNA-binding proteins. This enables them to act as mediators of intercellular communication, delivering regulatory signals to distant cells and organs and contributing to a novel mechanism of cell–cell and cell–organ crosstalk. Importantly, the specific contribution of miRNAs to distinct cellular and physiological processes can be elucidated experimentally by deleting components of the miRNA biogenesis pathways or by chemically inhibiting miRNA processing enzymes, which in turn disrupts the generation and function of mature miRNAs. This approach has provided valuable insights into their roles in development, disease, and tissue homeostasis [21,22,36]. From a translational perspective, it is important to highlight the diagnostic and prognostic value of circulating miRNAs, particularly those detectable in easily accessible biofluids such as serum, plasma, or urine, making them highly suitable as non-invasive biomarkers. Altered expression patterns of specific circulating miRNAs have been linked to the onset, progression, and prognosis of a variety of diseases. Their ability to reflect organ-specific injury and dynamic changes in disease states underscores their potential for early detection, risk stratification, and treatment monitoring in clinical practice [36].

As a result, miRNAs play a fundamental role in regulating numerous essential aspects of cellular function and maintaining tissue homeostasis. They exert control over processes such as cell growth, proliferation, differentiation, metabolism, and programmed cell death (apoptosis). By fine-tuning the expression of target genes involved in these pathways, miRNAs contribute to the proper development and adaptation of cells to internal and external stimuli. Dysregulation of miRNA networks has been linked to a wide range of pathological conditions, including cancer, cardiovascular diseases, inflammatory disorders, and organ injury, underscoring their importance as key modulators of health and disease [37,38]. To date, researchers have identified around 3000 distinct human miRNAs, and it is estimated that these small regulatory RNAs collectively influence the expression of up to 30% of all human mRNA transcripts [39]. Because of their extensive reach, miRNAs are believed to participate in the regulation of virtually every major biological process, including development, immune responses, metabolism, stress adaptation, and tissue repair. Their pervasive impact on gene networks highlights their crucial role as master regulators of cellular behavior. As such, alterations in miRNA expression or function can have profound consequences, contributing to the onset and progression of numerous diseases. From a clinical and applied perspective, microRNAs (miRNAs) play crucial and specific roles in various organ systems, acting as key regulators of homeostasis and disease. In the kidney, for example, miRNAs have been identified as fundamental players in nephron physiology and the development of various kidney diseases [40]. Similarly, in the liver, the expression of specific miRNAs is closely correlated with pathologies such as non-alcoholic fatty liver disease (NAFLD) and fibrosis. MiR-122, the most abundant miRNA in the liver, is a prime example, with its serum levels reflecting hepatocellular integrity and damage [41,42]. In the nervous system, miRNAs are essential for neuronal development, synaptic plasticity, and cognitive function. The dysregulation of specific miRNAs, such as miR-21, has been linked to neurodegenerative diseases and brain injuries. Therefore, analyzing the expression profile of miRNAs in biological samples like urine or plasma not only offers diagnostic and prognostic potential for specific organ pathologies but also paves the way for the development of targeted therapies based on the modulation of these small gene regulators [43].

### 6.1. Technological Platforms for miRNA Profiling

From a technical standpoint, the study and quantification of miRNAs rely on a variety of cutting-edge technological platforms. Quantitative real-time PCR (qRT-PCR) remains the gold standard for validating specific miRNAs, offering exceptional sensitivity and specificity for measuring the expression of single miRNAs or small panels. Its primary advantage is its accuracy and ability to precisely quantify even low-concentration samples. However, for a broader investigation, such as profiling thousands of miRNAs, high-throughput technologies are essential. Microarrays provide an effective solution for simultaneously analyzing the expression of hundreds of known miRNAs. These chips contain specific probes that bind complementarily to miRNAs in the sample. Their strength lies in their speed and capacity to generate comprehensive expression profiles, which are highly valuable for large-scale screening studies. Next-Generation Sequencing (NGS), specifically small RNA sequencing (small RNA-seq), is now the preferred platform for comprehensive “miRNome” profiling. Unlike microarrays, which are limited to known miRNAs, NGS is an unbiased approach that allows for the identification and quantification of not only previously discovered miRNAs but also novel miRNAs and their different isoforms (isomiRs). The depth and resolution of sequencing provide an unprecedented wealth of data, which is essential for basic research and the discovery of new biomarkers [44,45,46,47].

### 6.2. The Integration of Bioinformatics and Functional Analysis

Beyond quantification, understanding the role of miRNAs requires an analysis of their interactions with target genes. This process would not be possible without bioinformatics. Computational approaches utilize specialized tools and databases to predict potential mRNA targets of miRNAs. Databases such as TargetScan, miRDB, and miRTarBase are based on algorithms that evaluate sequence complementarity between the miRNA and the 3′ untranslated region (3′ UTR) of the mRNA, in addition to the evolutionary conservation of the binding site. The validity of these predictions is often confirmed with laboratory experiments, such as reporter gene assays or protein analysis. The integration of experimental data (profiling with NGS or microarrays) and bioinformatic analysis is a crucial step. For instance, a set of miRNAs with altered expression in a disease can be analyzed with a prediction tool to identify their potential targets. Subsequently, pathway analysis software (such as DAVID or KEGG) can be used to discover which biological pathways are most influenced by these miRNAs. This synergy between profiling technology and computational tools is fundamental for transforming raw data into a functional understanding of pathological mechanisms and for accelerating the discovery of new therapeutic targets [45,48,49,50,51].

## 7. microRNAs in Acute Kidney Injury: Functions and Implications

MicroRNAs (miRNAs) play a crucial role in kidney development, homeostasis, and function by regulating gene expression at the post-transcriptional level. They are involved in key processes such as cellular differentiation, proliferation, apoptosis, and response to stress, ensuring proper formation and maintenance of renal structures. Additionally, miRNAs contribute to the fine-tuning of signaling pathways essential for nephron development, tubular function, and overall kidney health, and their dysregulation has been implicated in various kidney diseases and disorders [29,30,52]. Pavkovic et al. have reported the presence of 669 distinct microRNA species in healthy human kidney tissue [53], highlighting the extensive and complex regulatory network these small non-coding RNAs contribute to within the renal environment. Furthermore, recent studies have established that miRNAs serve as key modulators of multiple signaling pathways that play critical roles in the development and progression of kidney diseases, both in experimental models and clinical settings. Alterations in the normal expression patterns of miRNAs frequently lead to disruptions in cellular processes, which can drive the advancement of renal impairment and contribute to worsening kidney function. Moreover, notable resistance to enzymatic degradation in biofluids, such as plasma and urine, enhances their potential as dependable biomarkers for detecting pathological alterations within the kidney and beyond [54]. This resilience against degradation under various storage and handling conditions makes circulating and urinary miRNAs particularly attractive for non-invasive diagnostic applications.

In fact, numerous studies have explored the diagnostic and prognostic value of serum, plasma, and urinary miRNAs in a range of renal disorders, including hypertensive and diabetic nephropathy, nephrotic syndrome and IgA disease, CKD and AKI [55]. Their expression profiles often reflect underlying disease mechanisms, providing insight into both disease onset and progression.

More specifically, distinct miRNA molecules have been identified as pivotal players not only in the early detection of AKI but also in guiding therapeutic strategies. By modulating key molecular pathways involved in inflammation, cell death, and tissue repair, these miRNAs offer promising avenues for targeted intervention and personalized treatment approaches in AKI management (Table 2).

– Sepsis-induced AKI

MiR-21-5p has emerged as a significant contributor to the pathogenesis of sepsis-induced AKI [56]. This microRNA exerts its effects primarily by targeting and suppressing RUNX1 (runt-related transcription factor 1), a key regulator implicated in promoting inflammatory responses, apoptotic cell death, and oxidative stress within renal tissues. The upregulation of RUNX1 has been correlated with exacerbated kidney damage during sepsis, driven by increased levels of systemic inflammation and cellular injury. Experimental studies have demonstrated that administration of a miR-21-5p mimic can effectively downregulate RUNX1 expression, thereby attenuating inflammatory cascades and reducing apoptosis and oxidative stress in the kidneys. This therapeutic intervention has been associated with marked improvements in renal function, suggesting that miR-21-5p may hold promise as a potential target for mitigating sepsis-related AKI and preserving kidney integrity [56]. In addition to miR-21-5p, miR-374a-3p has also been implicated in the molecular mechanisms underlying sepsis-induced AKI. This microRNA exerts its pathogenic influence through interaction with the long non-coding RNA small nuclear RNA host gene 5 (SNHG5), which has been shown to promote renal cell apoptosis and enhance the release of pro-inflammatory cytokines. Elevated SNHG5 expression contributes to kidney injury by amplifying the inflammatory response and facilitating cell death [57]. Interestingly, downregulation of SNHG5—mediated by miR-374a-3p—has been associated with a suppression of nuclear factor kappa-light-chain-enhancer of activated B cells (NF-κB) signaling activity, a critical pathway involved in the transcription of inflammatory mediators. This regulatory effect is further modulated by the interaction of miR-374a-3p with toll-like receptor 4 (TLR4), suggesting a complex network in which miRNAs, lncRNAs, and immune receptors coordinate the inflammatory response during sepsis-induced kidney injury [57]. Moreover, elevated levels of miR-29a and miR-10a-5p have been observed in patients with sepsis-induced AKI, and their expression has been shown to correlate with improved clinical outcomes. Notably, both microRNAs have demonstrated potential as reliable prognostic biomarkers, being associated with better 28-day survival rates. These findings suggest that miR-29a and miR-10a-5p may not only reflect underlying protective or adaptive responses but also serve as valuable tools for risk stratification and outcome prediction in critically ill patients with septic AKI [58].

– Contrast-induced AKI

In the context of contrast-induced AKI, studies have documented that the expression levels of miR-30a, miR-30c, and miR-30e are significantly elevated—showing approximately a two-fold increase—compared with healthy controls. This upregulation has been observed in patients developing this multifactorial syndrome following exposure to contrast media. The marked rise in these specific miRNAs suggests their involvement in the cellular stress response and injury pathways triggered by contrast agents. Furthermore, their consistent elevation indicates that members of the miR-30 family may serve as promising early biomarkers for the detection and monitoring of contrast-induced nephropathy, potentially enabling more timely intervention to limit renal damage [59]. Furthermore, Shi-Qun Sun and colleagues reported that patients who developed AKI following coronary angiography or percutaneous coronary intervention exhibited significantly elevated levels of miR-30a, miR-30b, and miR-188 compared to matched control individuals who did not experience renal complications. This differential expression suggests that these microRNAs may play a role in early molecular events leading to kidney injury in the setting of contrast exposure. Their increased levels may reflect underlying processes such as tubular epithelial stress, inflammation, or endothelial dysfunction. Consequently, miR-30a, miR-30b, and miR-188 hold potential as predictive biomarkers for contrast-induced AKI in high-risk cardiovascular patients, providing a window for early detection and possible preventative strategies [60].

– Cardiac surgery-induced AKI

**Figure 2:**
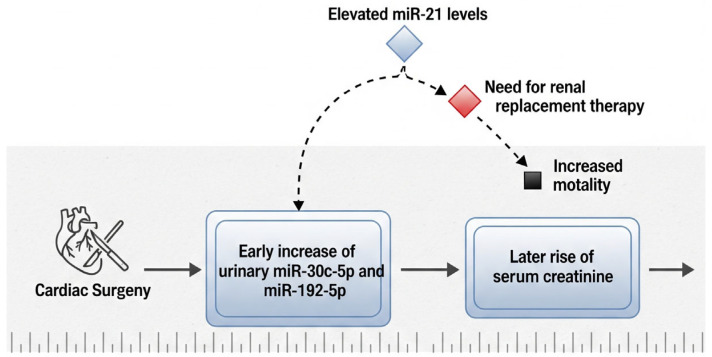
Dynamics of urinary and serum microRNAs in cardiac surgery–associated AKI. An early increase in urinary miR-30c-5p and miR-192-5p precedes the subsequent rise in serum creatinine. Elevated levels of miR-21 are associated with worse outcomes, including a higher risk of requiring renal replacement therapy and increased mortality.

In patients who develop AKI following cardiac surgery, significant elevations in urinary levels of miR-30c-5p and miR-192-5p have been observed as early as two hours after the surgical procedure. This early increase suggests a potential role for these microRNAs as sensitive biomarkers for the prompt prediction of postoperative renal injury, possibly preceding changes in conventional markers such as serum creatinine [61]. Their rapid response to renal stress underscores their clinical utility in enabling timely diagnosis and facilitating early intervention. Additionally, research by Du et al. has emphasized the prognostic value of both urinary and plasma miR-21 levels in the postoperative setting. Elevated miR-21 concentrations were found to be associated with an increased likelihood of requiring renal replacement therapy (RRT), extended hospitalization, and higher 30-day in-hospital mortality rates [62]. These findings support the integration of miRNA profiling into postoperative risk assessment models, as it may enhance clinicians’ ability to identify high-risk patients and guide decisions regarding intensive monitoring and early therapeutic strategies (Figure 2).

– Ischemia/reperfusion (I/R)-induced kidney injury

MicroRNAs have been shown to play a critical role in the molecular mechanisms underlying ischemia/reperfusion (I/R)-induced kidney injury. Their regulatory functions influence numerous cellular processes, including inflammation, oxidative stress, and apoptosis, which together contribute to the extent of renal damage during ischemic episodes. In particular, miR-330-5p is upregulated under the control of the long non-coding RNA 122049, which stimulates the expression of the ETS-domain transcription factor ELK1. This signaling pathway has been reported to attenuate renal cell apoptosis in the context of ischemic AKI by promoting protective gene expression programs [63]. The regulatory interaction between miR-330-5p and ELK1 highlights a promising therapeutic target for mitigating kidney injury caused by ischemia/reperfusion events [63]. Furthermore, the overexpression of miR-21 during renal ischemia/reperfusion has been proposed to exert anti-apoptotic effects, particularly in models of delayed ischemic preconditioning in mice [64]. Experimental evidence indicates that suppression of miR-21 leads to a marked increase in the expression of programmed cell death protein 4 (PDCD4), which in turn amplifies tubular epithelial cell apoptosis and aggravates kidney injury. These findings suggest that miR-21 acts as an endogenous protective factor, modulating cell survival pathways to limit tissue damage after ischemic insult [64]. Overall, targeting specific miRNA-mediated networks, such as the miR-330-5p/ELK1 axis and miR-21 signaling, may offer innovative therapeutic strategies to preserve renal function and improve outcomes in patients with ischemic AKI.

An experimental animal study investigating the p53/miR-17-5p/death receptor 6 (DR6) signaling axis in renal ischemia/reperfusion injury (IRI) demonstrated that miR-17-5p expression is significantly upregulated in renal tubular epithelial cells under hypoxic conditions. In vitro experiments further revealed that miR-17-5p directly targets and suppresses DR6, a receptor involved in apoptosis pathways, thereby reducing programmed cell death in hypoxic tubular cells. In vivo administration of miR-17-5p mimics resulted in marked downregulation of DR6 expression and conferred significant protection against IRI-induced kidney damage, highlighting the therapeutic potential of this microRNA in attenuating ischemic injury. Notably, the protective effects of miR-17-5p were shown to be critically dependent on p53 activity during ischemia/reperfusion, indicating that p53 functions as an upstream regulator modulating miR-17-5p induction. A time-course analysis conducted over a 48-h period demonstrated that miR-17-5p expression peaked approximately 24 h following the ischemic insult, suggesting a dynamic and time-sensitive regulatory role in the early phases of renal repair and adaptation after reperfusion. These findings provide compelling evidence that targeting the p53/miR-17-5p/DR6 pathway could represent a promising strategy to mitigate acute tubular injury and improve recovery following ischemic events [65].

Acute kidney injury (AKI) is widely recognized as a significant risk factor contributing to the progression of chronic kidney disease, as repeated or severe episodes of AKI can result in persistent structural damage and loss of renal function. In this context, the capacity for cellular proliferation and regeneration of tubular epithelial cells is essential to restore nephron integrity and recover kidney performance after injury. Recent research has demonstrated that miR-874-3p plays a protective role in this reparative process. Specifically, miR-874-3p has been shown to attenuate injury to renal tubular epithelial cells caused by cisplatin administration, a chemotherapeutic agent known for its nephrotoxic effects. By modulating key molecular pathways involved in apoptosis and inflammation, miR-874-3p contributes to preserving cell viability and maintaining tubular architecture during toxic insult. These findings highlight the therapeutic potential of targeting miR-874-3p as a strategy to counteract drug-induced kidney injury and to promote effective regeneration of renal tissue following AKI [66]. Additionally, in the setting of ischemia/reperfusion-induced kidney injury, miR-17 expression is markedly upregulated. This increase has been associated with enhanced proliferation of renal cells and facilitation of tissue repair processes, ultimately contributing to the recovery of kidney structure and function. In addition, miR-24, miR-126, miR-494 and miR-687 resulted in important actors for the regulation of inflammation, cell cycle and apoptosis in the repair stages of AKI [67].

– Renal Fibrosis

MiRNAs have emerged as crucial regulators in the pathophysiology of renal fibrosis, a key feature that drives the progression of virtually all chronic kidney diseases, independent of their initial etiology. Renal fibrosis is characterized by excessive deposition of extracellular matrix components, leading to scarring, disruption of normal tissue architecture, and irreversible loss of kidney function. MiRNAs influence this fibrotic process by modulating the expression of genes involved in inflammation, fibroblast activation, epithelial-to-mesenchymal transition (EMT), and matrix remodeling. Accumulating evidence from various animal models has demonstrated a pronounced upregulation of miR-21 in diabetic kidney disease, one of the most common causes of chronic kidney injury worldwide. MiR-21 has been shown to actively promote profibrotic signaling cascades, including the TGF-β pathway, which drives fibroblast proliferation and extracellular matrix synthesis, resulting in progressive tissue fibrosis. Importantly, experimental approaches involving the genetic deletion or pharmacological inhibition of miR-21 consistently reveal a significant reduction in renal fibrosis, preservation of normal kidney histology, and improved functional outcomes. These findings highlight miR-21 not only as a biomarker of disease progression but also as a promising therapeutic target. By specifically modulating miR-21 activity, it may be possible to interrupt the fibrotic remodeling process, thereby slowing or even halting the decline of renal function in patients with chronic kidney disease. Continued research into miRNA-based interventions holds great potential to revolutionize the management of fibrotic kidney diseases [68]. Specifically, the profibrotic actions of miR-21 are mediated through the downregulation of key regulatory molecules such as peroxisome proliferator-activated receptor alpha (PPARα) and phosphatase and tensin homolog (PTEN). By suppressing these targets, miR-21 promotes fibrogenic signaling pathways that contribute to fibroblast activation, extracellular matrix accumulation, and the progression of renal fibrosis [69]. A recent systematic review by Douvris et al. reported that miR-21 was the most commonly reported dysregulated microRNA and the most frequently implicated across all AKI categories, with upregulation observed in AKI due to nephrotoxins, kidney transplants, and other causes [29]. This widespread involvement underscores the central role of miR-21 in AKI pathophysiology and has drawn significant attention to its potential as both a biomarker and therapeutic target. Several studies have highlighted the protective role of miR-21 in the early stages of AKI, particularly in experimental models of sepsis-induced kidney injury, where administration of miR-21 mimics has been shown to attenuate inflammation, oxidative stress, and apoptosis. These effects suggest that miR-21 could serve as a promising therapeutic target in acute settings. However, its role is complex and appears to be both time- and context-dependent. While miR-21 upregulation may be adaptive and protective during the initial insult, a growing body of evidence has also consistently associated miR-21 with the activation of profibrotic signaling pathways during the recovery phase following injury. Sustained or prolonged expression of miR-21 may promote maladaptive repair processes, interstitial fibrosis, and ultimately contribute to the progression from AKI to chronic kidney disease (CKD). Therefore, any strategy aimed at modulating miR-21 expression must carefully consider not only the timing and duration of intervention, but also the underlying etiology and the specific cellular and molecular environment. A deeper understanding of the temporal dynamics and context-dependent effects of miR-21 will be essential to maximize its protective potential in the acute phase while minimizing the risk of fibrotic complications during recovery. Several studies have highlighted the protective role of miR-21 in the early stages of acute kidney injury (AKI), particularly in experimental models of sepsis-induced kidney injury, where administration of miR-21 mimics has been shown to attenuate inflammation, oxidative stress, and apoptosis. These effects suggest that miR-21 could serve as a promising therapeutic target in acute settings. However, its role is complex and appears to be both time- and context-dependent. Indeed, while miR-21 upregulation may be adaptive and protective during the initial insult, facilitating cell survival and limiting damage, a growing body of evidence has also consistently associated miR-21 with the activation of profibrotic signaling pathways during the recovery phase following injury. This dual function reflects a shift from beneficial to potentially harmful effects: sustained or prolonged miR-21 expression can promote maladaptive repair processes, leading to interstitial fibrosis and contributing to the progression from AKI to chronic kidney disease (CKD). Therefore, any therapeutic strategy targeting miR-21 must be finely tuned, considering not only the timing and duration of the intervention, but also the specific cellular and molecular microenvironment in which miR-21 acts. A better understanding of the regulatory mechanisms governing miR-21 expression and activity over time will be essential to harness its protective effects while minimizing fibrotic complications. This nuanced perspective may open the door to phase-specific or combination therapies that modulate miR-21 expression dynamically, maximizing benefit in the acute phase and curbing its deleterious consequences during recovery [29,40].

**Figure 3:**
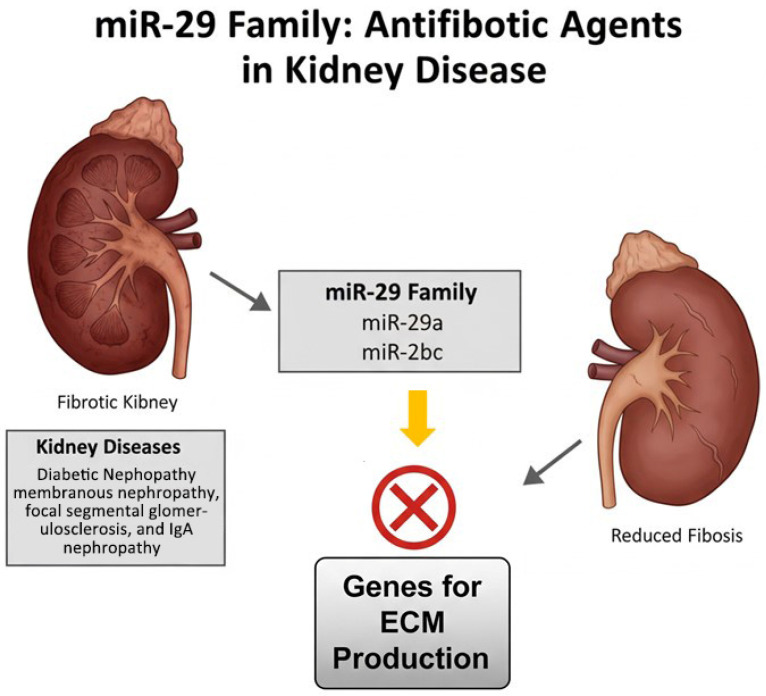
Antifibrotic role of the miR-29 family. The image illustrates the antifibrotic role of the miR-29 family in kidney disease: by inhibiting genes involved in extracellular matrix (ECM) production, miR-29 helps reduce fibrosis in conditions such as diabetic nephropathy, membranous nephropathy, focal segmental glomerulosclerosis, and IgA nephropathy.

Conversely, miR-29 family members—including miR-29a, miR-29b, and miR-29c—have been identified as potent antifibrotic agents in various kidney diseases such as diabetic nephropathy, membranous nephropathy, focal segmental glomerulosclerosis, and IgA nephropathy. These microRNAs help inhibit fibrotic processes by targeting genes involved in extracellular matrix production and deposition, thereby mitigating tissue scarring and preserving renal function [70] (Figure 3).

## 8. microRNAs and Extracellular Vesicles: Role, Communication and Biomarkers Potential

**Figure 4:**
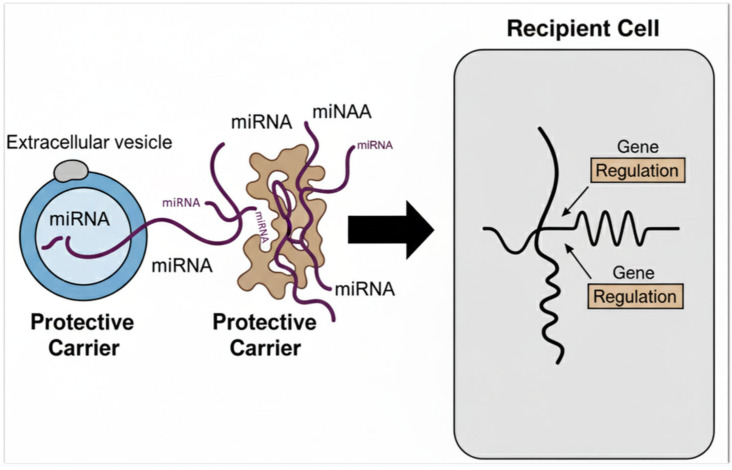
Protection and Transport of Extracellular MicroRNAs. This diagram illustrates the two main ways in which microRNAs (miRNAs) are protected from degradation and transported between cells. On the left, miRNAs are shown either encapsulated within an Extracellular vesicle, which acts as a protective shield, or bound to a Protein Carrier. These protected miRNAs are then transported to a Recipient Cell, where they are taken up. Once inside the cell, they play a crucial role in Gene Regulation by influencing the expression of target genes.

A remarkable discovery in the field of molecular biology is the presence of microRNAs (miRNAs) outside of cells, circulating in the extracellular environment and various biological fluids such as blood plasma, urine, saliva, and cerebrospinal fluid. Despite the abundant presence of ribonucleases (RNases)—enzymes that rapidly degrade RNA molecules—these extracellular miRNAs exhibit remarkable stability. This resilience is largely attributed to their association with protective carriers such as extracellular vesicles (including exosomes and microvesicles), lipoprotein complexes, or RNA-binding proteins. Their stable presence in bodily fluids not only challenges previous assumptions about RNA fragility but also opens up exciting possibilities for their use as minimally invasive biomarkers for a wide range of physiological and pathological conditions [71]. MicroRNAs are protected from degradation by RNases through several mechanisms, primarily their encapsulation within extracellular vesicles (EVs) such as microvesicles, exosomes, and apoptotic bodies. These membrane-bound vesicles provide a physical barrier that shields miRNAs from enzymatic breakdowns in the extracellular environment. Additionally, miRNAs can form stable complexes with RNA-binding proteins, such as Argonaute 2 (Ago2) and high-density lipoproteins (HDL), which further safeguard them from degradation. These protective strategies not only ensure the remarkable stability of circulating miRNAs in biological fluids but also facilitate their transport and functional delivery to recipient cells, enabling intercellular communication and regulation of gene expression across different tissues [71,72] (Figure 4). EVs are small, membrane-enclosed particles actively secreted by virtually all cell types into the extracellular space. These vesicles are surrounded by a lipid bilayer membrane that protects their cargo and allows for stable transport in biological fluids. Their size can vary considerably, typically ranging from about 30 nanometers, as seen with the smallest subtype known as exosomes, up to 2000 nanometers or more in larger vesicles such as apoptotic bodies. This size variability reflects differences in their cellular origin, biogenesis pathways, and functional roles, with smaller EVs generally originating from endosomal compartments and larger ones budding directly from the plasma membrane or released during programmed cell death [73]. These vesicles carry a diverse cargo that includes proteins involved in their biogenesis and intracellular trafficking, as well as specific cellular and tissue-derived molecules such as proteins, DNA, and RNA. Analyzing the molecular composition of EVs—both their proteomic and nucleic acid content—can provide valuable insights into the cell or tissue from which they originate, as well as their physiological or pathological state. In particular, exosomes and small non-coding RNAs contained within EVs have been implicated in the pathogenesis of kidney and heart diseases. They act as mediators of intercellular communication by delivering genetic material and signaling molecules to target cells, thereby influencing cellular functions and contributing to disease mechanisms. This capacity to transfer bioactive molecules highlights the potential role of EVs in modulating tissue responses and disease progression. Through these transfers, exosomes influence cellular signaling pathways, modulate gene expression, and alter the functional behavior of target cells. This dynamic exchange of molecular information contributes to disease pathophysiology by affecting processes like inflammation, fibrosis, apoptosis, and tissue remodeling. Understanding the mechanisms by which exosomes and their RNA content regulate cell-to-cell communication could uncover new biomarkers and therapeutic targets for renal disorders [71,72,73,74].

Microvesicles (MVs) facilitate the transfer of genetic information between cells through the delivery of messenger RNA (mRNA), microRNA (miRNA), and specific transcription factors. They accomplish this either by functioning as signaling complexes or by transferring surface receptor molecules to recipient cells. This mechanism enables the horizontal transfer of genetic material, which can induce epigenetic modifications in the target cells. For example, endothelial-derived MVS are capable of transferring mRNA that can alter the behavior and phenotype of recipient cells. The significance of this RNA transfer was highlighted by observations that treatment with RNase, an enzyme that degrades RNA, can inhibit the biological effects associated with MVs, thereby confirming that RNA cargo plays a crucial role in mediating these intercellular communications [73]. Several studies have demonstrated that MVs derived from stem cells transport not only messenger RNA (mRNA) but also miRNAs, which possess the capacity to silence specific genes and regulate protein synthesis [75]. In recent years, urinary exosomes have emerged as promising non-invasive biomarkers for a variety of kidney diseases. For instance, elevated levels of fetuin-A in urinary exosomes have been identified as an early indicator of cisplatin-induced nephrotoxicity, particularly in patients with AKI admitted to intensive care units. Additionally, experimental models in rats have revealed that decreased expression of aquaporin-1 within urinary exosomes correlates with ischemia–reperfusion (IR) injury in the kidneys, highlighting its potential utility as a marker of renal damage [73].

MiRNAs demonstrate notable stability in various extracellular fluids, including blood, sweat, and urine. This resilience is largely attributed to their encapsulation within MVs and other EVs, which shield them from direct degradation by RNases. The ability of EVs to mediate the transfer of miRNAs between cells also provides a mechanism that contributes to processes such as tumor metastasis, by enabling the communication and genetic influence between cancer cells. Moreover, miRNAs can evade RNase-mediated breakdown by forming protective complexes with specific RNA-binding proteins. Interestingly, circulating levels of RNases are influenced by kidney function, suggesting that alterations in renal activity could impact RNase concentrations in the bloodstream. Consequently, changes in renal function may modulate the degradation rate of circulating miRNAs, highlighting a potential link between kidney health and miRNA stability [72,73].

## 9. Toward miRNA-Based Precision Medicine in Acute Kidney Injury

In recent years, multiple biomarkers have emerged to enhance the early detection and monitoring of AKI, addressing the limitations of traditional markers like serum creatinine. Serum creatinine remains the cornerstone for AKI diagnosis but is hampered by significant drawbacks: it rises slowly, often lagging behind actual renal injury by 24–36 h, and is influenced by variables such as muscle mass, hydration status, and hemodynamics—factors especially problematic in critically ill patients—resulting in poor sensitivity and specificity [76,77]. In contrast, biomarkers such as NGAL, KIM-1, L-FABP, IL-18, TIMP-2 × IGFBP-7, and others have shown promise for earlier and more accurate detection. However, the utility of these biomarkers is not uniform across all patient populations. While these markers provide valuable clinical information, they often lack sufficient sensitivity and specificity across different patient populations and clinical contexts [78,79]. In this regard, the integration of miRNA research into AKI management holds great promise for advancing precision medicine. By identifying specific miRNA signatures linked to the early stages and progression of AKI, clinicians could achieve earlier diagnosis with greater accuracy than current biomarkers allow. This improved molecular profiling enables the stratification of patients based on their individual miRNA expression patterns, facilitating tailored therapeutic strategies that address the unique pathophysiological mechanisms driving their kidney injury [80]. Furthermore, targeting dysregulated miRNAs involved in inflammation, cell death, and tissue repair offers a novel therapeutic avenue to halt or even reverse kidney damage, moving beyond the one-size-fits-all approach. Rather than replacing established biomarkers, miRNA-based diagnostics could integrate with them, potentially improving risk stratification, enabling earlier detection, and supporting a more personalized approach to AKI management. Such personalized interventions could optimize treatment efficacy while minimizing side effects, ultimately improving patient outcomes. Continued research and clinical validation are essential to translate these molecular insights into practical diagnostic tools and targeted therapies, paving the way for a new era in AKI care that combines precision diagnostics with individualized treatment plans, thereby reducing the overall burden of this serious condition [81].

## 10. Therapeutic Potential of miRNAs in AKI

Beyond their role as biomarkers, microRNAs are increasingly being investigated as therapeutic targets in AKI. Recent clinical data suggest that therapeutic modulation of miRNAs holds promise in AKI Antagomirs, designed to silence pathogenic miRNAs, and miRNA mimics, aimed at restoring protective ones, represent innovative strategies to translate biological insights into clinical applications. For instance, targeting miRNAs involved in inflammation, apoptosis, and fibrosis could mitigate tubular damage and promote renal recovery. Integrating these approaches with conventional AKI management may help bridge the gap between mechanistic understanding and effective therapies, paving the way for precision medicine in nephrology [82]. Dysregulated miRNAs such as miR-21, miR-24, and miR-494 have been implicated in key pathological pathways, including inflammation, apoptosis, oxidative stress, and fibrosis. Preclinical studies have demonstrated that experimental approaches using antagomirs (miRNA inhibitors) or miRNA mimics can restore physiological gene regulation, thereby reducing tubular epithelial cell death, attenuating inflammatory responses, and promoting tissue repair. For example, inhibition of miR-21 has been shown to reduce fibrosis and maladaptive repair [83], while targeting miR-24 can limit necroptosis and tubular injury [84,85]. Similarly, modulation of miR-494 influences mitochondrial apoptosis pathways and cell survival [86]. These findings suggest that therapeutic modulation of specific miRNAs could move clinical management of AKI beyond conventional supportive care, offering targeted molecular interventions that address the underlying mechanisms of renal injury. However, important challenges remain, including the need for safe and efficient delivery systems, minimization of off-target effects, and validation of efficacy in clinically relevant models. As research progresses, combining miRNA-based therapies with precision diagnostics may open the door to personalized nephrology, where interventions are tailored according to a patient’s specific miRNA signature. Such strategies hold the potential to not only prevent progression of AKI but also promote functional recovery and reduce the transition to chronic kidney disease. In recent years, innovative delivery platforms have been explored to enhance the therapeutic potential of miRNA-based interventions in renal disease. Traditional approaches to miRNA modulation often suffer from limited tissue specificity and the risk of off-target effects, which may reduce efficacy and raise safety concerns. To address these limitations, several emerging strategies have been proposed. Extracellular vesicles, for instance, represent natural carriers capable of transferring nucleic acids and proteins between cells, thereby offering a biocompatible and targeted system for miRNA delivery. Similarly, nanoparticles have been engineered to encapsulate and protect therapeutic miRNAs from degradation while enabling controlled release at the site of injury. More recently, engineered exosomes have gained attention as highly versatile vehicles, as they can be tailored to selectively target dysregulated miRNAs within renal tissue with remarkable precision. Collectively, these platforms hold promise not only in improving therapeutic efficiency but also in minimizing off-target interactions, paving the way for a new generation of personalized and safe miRNA-based therapies in acute and chronic kidney injury [87,88,89,90].

## 11. Current Limitations of miRNA Research in AKI

Despite the encouraging potential of miRNAs in AKI, several significant limitations must be acknowledged before these molecular insights can be fully integrated into daily clinical practice. Current gaps in the literature include the relatively small number of translational studies and the lack of standardized methodologies for detecting and quantifying miRNAs across different laboratories and clinical settings, which hampers reproducibility and widespread adoption. Furthermore, most findings have been generated in preclinical or highly selected patient cohorts, limiting their generalizability and underscoring the pressing need for larger, more diverse population studies. The validation of candidate miRNAs as reliable biomarkers will not only require these broader studies but also significant efforts to define robust reference ranges and develop reliable assay platforms. Finally, a substantial hurdle remains in translating miRNA-based therapies from research to the bedside, particularly regarding the development of safe and efficient delivery systems that can mitigate potential off-target effects and ensure clinical efficacy. In recent years, machine learning and bioinformatics approaches have become increasingly important for analyzing miRNA expression patterns in AKI. These tools enable the identification of complex molecular signatures, allowing patient stratification based on individual miRNA profiles and improving the prediction of disease onset, severity, and prognosis. Integrating such computational approaches with experimental and clinical data can enhance biomarker discovery, optimize risk assessment, and potentially guide personalized therapeutic interventions, moving toward precision medicine in AKI management. The application of machine learning and bioinformatics approaches to analyze large-scale miRNA datasets are significant limitations. While these computational tools can enhance biomarker discovery and patient stratification, they require specialized expertise and dedicated personnel. Not all centers have the necessary infrastructure or trained staff to implement such analyses effectively, which can hinder the reproducibility and widespread adoption of these advanced approaches. Finally, while preclinical data suggest therapeutic potential through modulation of specific miRNAs, the translation of these approaches into safe and effective clinical interventions remains an ongoing challenge.

## 12. Conclusions: Future Perspectives on miRNAs

In recent years, microRNAs have emerged as key regulators of gene expression involved in the pathogenesis of AKI, including pathways related to inflammation, apoptosis, oxidative stress, and fibrosis. Accumulating evidence supports their utility as sensitive and specific biomarkers for early detection, prognosis, and potentially even as therapeutic targets.

Despite the substantial body of research dedicated to miRNAs, there remains a clear and pressing need for further comprehensive investigation into their potential applications in both the diagnosis and treatment of AKI. Moreover, the therapeutic modulation of miRNAs offers promising development for innovative treatments aimed at halting or reversing the progression of AKI. By selectively targeting miRNAs involved in apoptotic pathways, inflammation, fibrosis, and cellular regeneration, it may be possible to develop novel strategies that not only protect renal tissue but also promote repair and functional recovery.

Ultimately, advancing research in this area holds the potential to revolutionize patient care by providing reliable, minimally invasive diagnostic tools—such as circulating miRNA signatures detectable in blood or urine—and paving the way for personalized medicine approaches tailored to individual molecular profiles. Given the high morbidity and mortality rates associated with AKI worldwide, these developments could substantially improve clinical outcomes, reduce healthcare costs, and enhance quality of life for affected patients. Continued multidisciplinary efforts integrating molecular biology, clinical research, and translational medicine will be essential to fully realize the promise of miRNA-based diagnostics and therapeutics in acute kidney injury.

## Figures and Tables

**Table 1:** Non-coding RNA Overview.

Type	Size	Function	Description
MicroRNA (miRNA)	20–24 nt	Gene expression regulation (post-transcriptional silencing)	Small RNAs that bind mRNA to inhibit translation or cause degradation
Small interfering RNA (siRNA)	20–25 nt	RNA interference (gene silencing)	Derived from double-stranded RNA, guides degradation of complementary mRNA
Piwi-interacting RNA (piRNA)	24–31 nt	Transposon silencing in germ cells	Interacts with Piwi proteins to silence transposable elements
Small nucleolar RNA (snoRNA)	60–300 nt	RNA modifications	Guides chemical modifications on other RNAs, mostly in the nucleolus
Long non-coding RNA (lncRNA)	>200 nt	Gene regulation, chromatin remodeling	Involved in diverse regulatory functions including transcriptional regulation
Ribosomal RNA (rRNA)	120–5000 nt	Structural and catalytic component of ribosomes	Forms the core of ribosome’s structure and catalyzes protein synthesis
Transfer RNA (tRNA)	73–95 nt	Carries amino acid during translation	Matches mRNA codons to the correct amino acid during protein synthesis

**Table 2:** The significance of miRNAs in acute kidney injury.

MiRNA	Effect	Origin of Sample	Study	Reference
Sepsis-induced AKI				
miR-21-5p	Reduction in systemic inflammation, cell apoptosis, and oxidative stress mediated by RUNX1	Serum and kidney tissue	Experimental	Zhang et al., 2021 [56]
miR-374a-3p	Reduction in apoptosis and cytokine secretion via suppression of SNHG5 expression along the TLR4/NF-κB pathway	Serum	Clinical	Wang et al., 2021 [57]
miR-29a and -10a-5p	Accurate markers for 28-day survival outcomes	Serum	Clinical	Huo et al., 2017 [58]
Contrast-induced AKI				
miR-30a, -30c, and -30e	More than a twofold elevation in patients with contrast-induced AKI cases relative to the control group	Plasma and kidney tissue	Experimental	Gutiérrez-Escolano et al., 2015 [59]
miR-188, -30 and -30e	Contrast-induced AKI patients exhibited over a 1.5-fold rise relative to controls	Plasma and kidney tissue	Clinical	Sun et al., 2016 [60]
Post-operative AKI				
miR-30c-5p and -192-5p	Marked elevation in urinary levels 2 h post-cardiac surgery	Urine	Clinical	Zou et al., 2017 [61]
miR-21	Elevated urine and plasma levels are linked to the onset and progression of AKI, and can predict the requirement for postoperative renal replacement therapy, 30-day in-hospital mortality, and prolonged hospitalization or ICU stay	Urine and plasma	Clinical	Du et al., 2013 [62]
I/R injury				
miR-330-5p	Decreased renal cell apoptosis during ischemic AKI, promoted by long non-coding RNA 122049	Serum and kidney tissue	In vito	Xiao et al., 2022 [63]
miR-21	Protective anti-apoptotic effect during delayed ischemic preconditioning in mice models	Serum and kidney tissue	Experimental	Xu et al., 2012 [64]
miR-17-5p	During hypoxia, suppression of death receptor 6 (DR6) leads to an antiapoptotic response	Kidney tissue	Experimental and in vitro	Hao et al., 2018 [65]
Cisplatin induced AKI				
miR-874-3p	Attenuation of renal tubular epithelial cell injury and enhancement of repair mechanisms after kidney injury	Serum and kidney tissue	In vitro	Yu et al., 2023 [66]
AKI				
miR-24, -126, -494 and -687	Regulation of inflammation, cell cycle and programmed cell death in the repair stages of AKI	Serum	Review	Ren et al., 2018 [67]
AKI progression				
miR-21	Its upregulation promotes renal fibrosis through the suppression of peroxisome proliferator-activator receptor alpha and phosphatase and tensin homolog	Serum	Review, clinical	Lv et al., 2018 [68], McClelland et al., 2015 [69]
Renal fibrosis in diabetic nephropathy				
miR-29a, -29b and -29c	Antifibrotic effect	Kidney tissue	Experimental	Wang et al., 2012 [70]

## Data Availability

No new data were created or analyzed in this study.

## Abbreviations

IRI—Ischemia–reperfusion injury; CKD—Chronic kidney disease; AKI—Acute kidney injury; miRNA—micro RNA; sn-RNA—small nuclear RNA; sno-RNA—small nucleolar RNA; rasi-RNA—Repeat associated small interfering RNA; sco-RNA—Small cell osteosarcoma RNA; si-RNA—small interfering; piRNA—Piwi-interacting RNA; ti-RNA—transcription initiation RNA; IR—Ischemia–reperfusion; MVs—Microvesicles; EVs—Extracellular vesicles.
